# Physiological Responses During Hybrid BNCI Control of an Upper-Limb Exoskeleton

**DOI:** 10.3390/s19224931

**Published:** 2019-11-12

**Authors:** Francisco J. Badesa, Jorge A. Diez, Jose Maria Catalan, Emilio Trigili, Francesca Cordella, Marius Nann, Simona Crea, Surjo R. Soekadar, Loredana Zollo, Nicola Vitiello, Nicolas Garcia-Aracil

**Affiliations:** 1 Miguel Hernández University of Elche, Av. Universidad w/n, Ed. Innova, 03202 Alicante, Spain; jcatalan@umh.es (J.M.C.); nicolas.garcia@umh.es (N.G.-A.); 2 Universidad de Cádiz, Av. de la Universidad n10, 11519 Puerto Real, Spain; 3 The BioRobotics Institute, Scuola Superiore Sant’Anna, Viale Rinaldo Piaggio 34, 56025 Pontedera, Pisa, Italy; e.trigili@santannapisa.it (E.T.); s.crea@santannapisa.it (S.C.); nicola.vitiello@santannapisa.it (N.V.); 4 Clinical Neurotechnology Laboratory, Department of Psychiatry and Psychotherapy (CCM), Charité-Universitätsmedizin Berlin, Charitéplatz 1, 10117 Berlin, Germany; surjo.soekadar@charite.de; 5 Unit of Advanced Robotics and Human-centred Technologies, Campus Bio-Medico University of Rome, 00128 Rome, Italy; f.cordella@unicampus.it (F.C.); L.Zollo@unicampus.it (L.Z.); 6 Applied Neurotechnology Laboratory, Department of Psychiatry and Psychotherapy, University Hopsital of Tübingen, Calwerstr. 14, 72076 Tübingen, Germany; marius.nann@uni-tuebingen.de; 7 IRCCS Fondazione Don Carlo Gnocchi, Via Alfonso Capecelatro 66, 20148 Milan, Italy; 8 New technologies for Neurorehabilitation Lab., Av. de la Hospitalidad, s/n, 28054 Madrid, Spain; 9 Department of Excellence in Robotics & AI, Scuola Superiore Sant’Anna, 56025 Pontedera, Pisa, Italy

**Keywords:** Assistive technologies, exoskeleton, brain-computer interfaces

## Abstract

When combined with assistive robotic devices, such as wearable robotics, brain/neural-computer interfaces (BNCI) have the potential to restore the capabilities of handicapped people to carry out activities of daily living. To improve applicability of such systems, workload and stress should be reduced to a minimal level. Here, we investigated the user’s physiological reactions during the exhaustive use of the interfaces of a hybrid control interface. Eleven BNCI-naive healthy volunteers participated in the experiments. All participants sat in a comfortable chair in front of a desk and wore a whole-arm exoskeleton as well as wearable devices for monitoring physiological, electroencephalographic (EEG) and electrooculographic (EoG) signals. The experimental protocol consisted of three phases: (i) Set-up, calibration and BNCI training; (ii) Familiarization phase; and (iii) Experimental phase during which each subject had to perform EEG and EoG tasks. After completing each task, the NASA-TLX questionnaire and self-assessment manikin (SAM) were completed by the user. We found significant differences (*p*-value < 0.05) in heart rate variability (HRV) and skin conductance level (SCL) between participants during the use of the two different biosignal modalities (EEG, EoG) of the BNCI. This indicates that EEG control is associated with a higher level of stress (associated with a decrease in HRV) and mental work load (associated with a higher level of SCL) when compared to EoG control. In addition, HRV and SCL modulations correlated with the subject’s workload perception and emotional responses assessed through NASA-TLX questionnaires and SAM.

## 1. Introduction

Around 80 million people in the EU, a sixth of its population, have a disability. They are often hindered from full social and economic participation by various barriers related to physical, psychological and social factors. Over 30% of people above the age of 75 are impaired to some extent, and over 20% are severely impaired. The percentage of people with disabilities is set to rise as the EU population ages [1].

Using brain-machine interfaces (BMIs) or brain-computer interfaces (BCIs) in combination with assistive robotic devices, such as wearable robots, has the potential to augment the capabilities of disabled people to carry out activities of daily living with success. Recent developments of BMIs or BCIs allow detection and translation of electric, magnetic or metabolic activity into control signals of external devices or machines. However, reliability and safety of these systems are insufficient for daily life applications. Thus, fusion of different bio-signals under the concept of brain/neural-computer interaction (BNCI) systems, also referred as hybrid BCIs or multimodal BCIs. They were introduced for the first time by [2].

The different BNCI approaches can be distinguished accordingly with the type of bio-signals used as inputs in the control of assistive or rehabilitation devices [3]. The first approach is based on the combination of different brain signals, such as Event-related Potential (ERP) and sensorimotor rhythm (SMR) induced by Motor Imagery (MI) [4], ERP and Steady State Evoked Potential (SSEP) [5,6,7], and SMR and SSEP [8,9]. The second approach is based on the fusion or combination of brain signals and other kind of bio-signals, such as sEMG [10,11], ECG [12,13] and electrooculography (EoG) [14,15].

On the other hand, there are already published some examples of multi-modal architectures for the interaction and control of upper-limb robotic exoskeletons. Specifically, Frisoli et al. presented a robotic-assisted rehabilitation training with an upper limb robotic exoskeleton for the restoration of motor function in spatial reaching movements [16]. Then, they presented a multimodal control of an arm-hand robotic exoskeleton to perform activities of daily living. The presented system was driven by a shared control architecture using BCI and eye gaze tracking for the control of an arm exoskeleton and a hand exoskeleton for reaching and grasping/releasing cylindrical objects of different size in the framework of the BRAVO project [17]. Moreover, Pedrocchi et al. developed a novelsystem composed by a passive arm exoskeleton, robotic hand orthesis and a neuro muscular electrical stimulation system driven by residual voluntary muscular activation, head/eye motion, and brain signals in the framework of the MUNDUS project [18].

**Figure 1:**
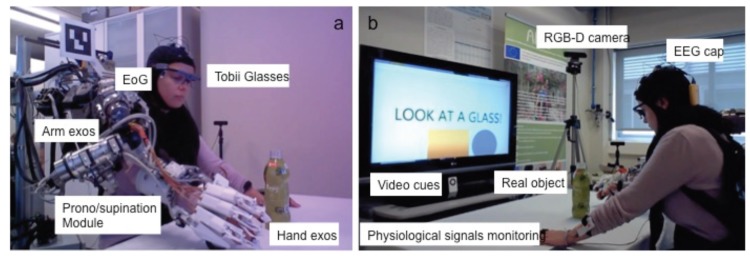
Experimental setup: All the assistive technology shown in the figure was developed in the AIDE EU project framework to assist arm and hand function after severe paralysis. (**a**): Wearable eye tracker (Tobbi Glasses), electrooculography (EoG) interface, arm exoskeleton including pronosupination module and hand exoskelton. (**b**): A context-sensitive 3D-camera and visual feedback support precise and reliable assistance to grasp a real object; EEG and physiological signals monitoring system.

Differently from the previous works, the multimodal approach, developed inside the AIDE European project, is based on a hybrid control interface based on the combination of a gaze tracking, electroencephalography (EEG) and EoG system to intuitively control an arm/hand exoskeleton for assisting in reaching and grasping objects to people with disabilities (see Figure 1). The AIDE multimodal control interface predicts the activity user wants to perform and allows the user to trigger the execution of different sub-actions that compose the predicted activity, and to interrupt the task at any time by means of the hybrid control interface.

The user’s psychological state is related to the use and performance in brain-computer interface seems to be a well accepted statement, but there are not so many studies to corroborate it. Kaufmann et al. in [19] studied correlations between P300-BCI based task performance and resting heart rate variability, reporting that frequency domain measures of heart rate variability were significantly associated with BCI-performance. Moreover, the correlation between the use BCI and fatigue, frustration and attention were investigated in [20,21], indicating that mental state was closely related to BCI use and performance; in [22], discriminating four levels of attention which can be used for boosting the BCI performance;and in [23], classifying three attention levels using a KNN classifier based on the Self-Assessment Manikin (SAM) model.

There are currently two use scenarios for BNCI systems: brain/neural control of assistive devices, such as wearable robots and prostheses, and use of BNCI systems for rehabilitation [24,25]. In the context of the first use scenario, involving EEG-signals, typically related to imagined or attempted actions, offers the advantage of intuitive control. Here, combination with EOG signals can improve real-world applicability of brain controlled devices, because EOG control is very accessible and can provide a reliable veto-function [26]. At the same time, EEG control can be cumbersome, particularly when the classification accuracy is rather low (e.g., due to low signal quality/artefacts) or when the user has not yet gained stable BNCI control. In the context of the second scenario, in contrast, brain signals are used to induce neuroplasticity triggering neural recovery [25]. Recently, it was suggested to combine both approaches, the assistive and restorative approach, to increase user acceptability and broad adoption of BNCI-based rehabilitation [27]. For this purpose, EEG/EOG brain/neural control has to be optimized in terms of mental work load and user engagement accessible through the subject’s physiological responses. Assessment of physiological responses during EEG/EOG brain/neural control would not only allow for designing optimal control strategies, but may also inform adaptation of the BNCI system during actual use (e.g., by providing feedback about the optimal window of operation, adapting the bio-signal detection thresholds etc.). In this paper, the user’s physiological reactions related to an exhaustive use of the interfaces in the brain/neural control of an assistive wearable arm/hand exoskeleton is analysed in two ways: in terms of the evolution and changes during a specific task; and the differences between the use of two different interfaces (EEG and EoG).

## 2. Materials and Methods

### 2.1. Multimodal Sensory System

All participants were sitting in a conformable chair in front of a desk and wore a whole-arm exoskeleton as well as wearable devices for monitoring physiological, EEG and EoG signals (Figure 1).

#### 2.1.1. Hybrid Bnci

**Figure 2:**
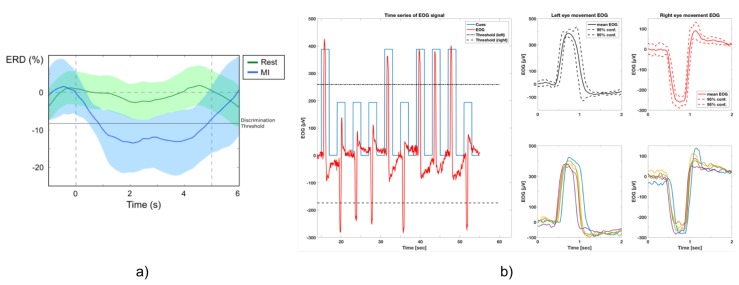
(Example of ERD and EOG control signals. (**a**) Example of event-related desynchronization (ERD) of “rest” and “motor imagery” (MI) phase. During calibration, discrimination threshold for reliable differentiation is set. (**b**) Overview of EOG control signals. Left side: EOG time series of horizontal eye movements (red). Movements are cued to left (blue long bars) and right (blue short bars) movements. Black dotted lines represent 60% discrimination thresholds based on maximum EOG peaks. Right side: Time-locked EOG signals of left/right horizontal eye movements during cue presentations. Plots show mean EOG with 95% confidence intervals (upper figures), which is calculated out of the EOG raw signals (lower figures).

The hybrid BNCI interface was used to control a vision-guided autonomous whole-arm exoskeleton to perform reaching and grassping tasks. The shared-human control of the whole-arm exoskeleton was implemented using a finite-state machine (FSM) triggered by electroencephalography/electrooculography (EEG/EOG) signals similar to the presented in [28]. Specifically, the reaching task was controlled by an EOG signal related to horizontal oculoversions (HOV) to the right (HOVr) and the hand closing task was controlled by computing the user intention through motor imagery-related EEG desynchronization of sensorimotor rhythms (SMR-ERD also termed μ-rhythm; 8 to 15 Hz) [26]. The SMR-ERD was computed using the power method proposed by Pfurtscheller and Lopes da Silva [29]. The EEG/EOG singals were recorded using the Enobio 8 (Neuroelectrics, Barcelona, Spain), an eight-channel wireless EEG recording system. The EEG signals were recorded from 5 electrodes placed at F3, T3, C3, Cz and P3 locations according to the 10–20 international system with ground and reference electrodes at AFz and FCz respectively. The last channel of Enobio system was used to detect the voluntary horizontal oculoversions (HOV) using EoG signals recorded from the left outer canthus referenced to left mastoid. Instead of conventional electrolyte gel electrodes, polyamide-based dry electrodes were used [30]. These electrodes might be a viable solution to substantially improve the cost-benefit ratio of wearing comfort, preparation time, user-friendliness, long-term stability, reliability and financial costs. EEG signals was recorded at a sampling rate of 200 Hz, band-pass filtered at 0.4–70 Hz and pre-processed using a Hjorth Laplacian filter centered on C3 electrode to attenuate line noise and movement artefacts [31]. Similar to EEG signal processing, EOG signals was recorded at a sampling rate of 200 Hz and pre-processed using a band-pass filtered at 0.1–30 Hz. A customized version of the open-source BCI2000 were used to calibrate and to translate user’s biosignals into control signals of the vision-guided autonomous whole-arm exoskeleton [26]. During the calibration, the following key features were computed: (i) the reference value (RV) of SMR-ERD related to externally paced intended grasping movements; (ii) the optimal frequency for SMR-ERD detection; (iii) the SMR-ED detection threshold; and (iv) the HOV detection threshold (Figure 2).

#### 2.1.2. Physiological Monitoring System

The physiological monitoring system acquires and processes signals, such as, electrocardiography (ECG), galvanic skin response (GSR) and respiratory plethysmography.

The selection of components of the sensory system for the physiological monitoring system was based on the requirements imposed by its application to the robotic assistance in daily life activities. Therefore, all devices need to be wearable and wireless, preferably directly on-body with combined sensor, processing and sending unit not to disturb users through connection or power cables during daily life tasks.

BioHarness 3 and Shimmer 3 GSR+ Unit, are wearable, wireless and provide a high wearing comfort to monitor ECG, respiratory plethysmography and GSR. Both devices have a built-in signal-processing unit sending the resulting information to the main processing unit via Bluetooth where further processing is performed.

The BioHarness 3 is a compact physiological monitoring device developed by Zephyr Technology. The device is based on a wearable strap that is placed around the chest. The strap contains the actual physiological sensors and a computing unit with Bluetooth module. Supported recording techniques are ECG, respiratory plethysmographie, skin temperature and acceleration measurement. This means that the BioHarness 3 can output the requested heart rate (HR), respiration rate (RR) and heart rate variability (HRV) which is a measure of the variation in time between each heartbeat. In particular, the SDANN is used as a feature of HRV, which is defined as the standard deviation of the average instantaneous heart rate intervals (NN) calculated over short periods. In our case, the SDANN is computed over a moving window of 300 s.

The Shimmer3 GSR+ Unit measures skin conductivity between two reusable electrodes mounted to two fingers of one hand. The output measure is the galvanic skin response (GSR). GSR is a common measure in psychophysiological paradigms and therefore often used in affective state detection.

### 2.2. Arm Exoskeleton

The arm exoskeleton can be splitted into two main parts: (i) a hand-wrist exoskeleton; and (ii) a shoulder-elbow exoskeleton.

#### 2.2.1. Shoulder-Elbow Exoskeleton

The shoulder-elbow exoskeleton (NESM) has four active degrees of freedom, namely shoulder adduction/abduction, flexion/extension and intra/extra rotation and elbow flexion/extension, together with eight additional passive degrees of freedom for the alignment of the motor axes to the human joint axes, regardless the user’s specific anthropometry sizes [32,33].

The main novelty introduced by this system relies on its mechanical architecture: the actuation units of the NESM are based on a Series Elastic Actuation (SEA) architecture. SEAs reduce the mechanical stiffness of the actuator and are easy to control in position mode and torque modes. Moreover, simple software algorithms can be implemented to monitor the torque delivered at each joint and avoid exceeding specific values, as well as detecting collisions with external objects.

#### 2.2.2. Hand-Wrist Exoskeleton

The hand-wrist exoskeleton is composed of two modules, the hand and the wrist, that can be used separately or in combination. The hand exoskeleton has three active degrees-of-freedom, corresponding to flexion-extension of index finger, flexion-extension of middle finger and flexion-extension of both ring and little finger. The thumb will have a series of passive-degrees of freedom that will allow placing the thumb in a suitable pose in the installation phase, and will be lockable to allow the thumb to work in opposition during the grasp [34].

The wrist exoskeleton was designed to be easily connected to the NESM exoskeleton.

### 2.3. Participants

Eleven BNCI-naive volunteers participated in the experiments(six males and five females). None of the study participants had any prior experience in the use of these kind of interfaces. All were healthy, without cognitive or physical deficits. They were aged between 26 and 42 (mean age 31 years, median age 29 years, standard deviation 6.3 years). Before inclusion, participants gave their written informed consent, including photography and video, and agreed that this material can be used in journals and other public media. The study protocol was approved by the local ethics committee of the Miguel Hernandez University (Elche, Spain).

### 2.4. Experimental Protocol

The experimental protocol consists of three phases:

1. **Set-up, calibration and BNCI training:** Calibration of the BNCI system comprises two parts: in the first part, participants are instructed to either relax or imagine hand-grasping motions following a visual cue displayed on a computer screen. To identify the optimal frequency for detection of motor-imagery related desynchronization of sensorimotor rhythms (SMR, 8–15Hz) of the subject, a power spectrum estimation is performed, selecting the frequency that shows largest even-related desynchronization (ERD) during motor-imagery and event-related synchronization (ERS) during relax. Based on the maximum values for ERD and ERS, an optimal discrimination threshold is computed and used for later online BCI control. EoG is recorded in accordance to the standard EoG placements at the left and right outer canthus (LOC/ROC). In the EoG-related part of the calibration, subjects are instructed to move their eyes to the left or to the right following randomized visual cues (arrow to the left, arrow to the right). A detection threshold for full left and right eye movements is set at 80% of the average of maximal EoG signal recorded during presentation of the visual cues.
2. **Familiarization:** The familiarization phase only consisted of showing the user the functioning of the finite-state machine and the visual interface. No additional training was required, since the user was already familiarized with the EEG/EoG interface.
3. **Experimental phase:** Each subject had to perform two different tasks during 6 min each one: (i) a task triggered by the EoG interface to reach an object controlling the arm exoskeleton; (ii) a task triggered by the EEG interface to grasp an object controlling the hand exoskeleton. To increase the number of data points for the analysis, subjects were asked to perform two times the EoG task and four times the EEG task. After completing each task with one interface, the NASA-TLX questionnaire and self-assessment manikin (SAM) were submitted to the user to evaluate the subjective workload required to perform the task.

## 3. Results

**Figure 3:**
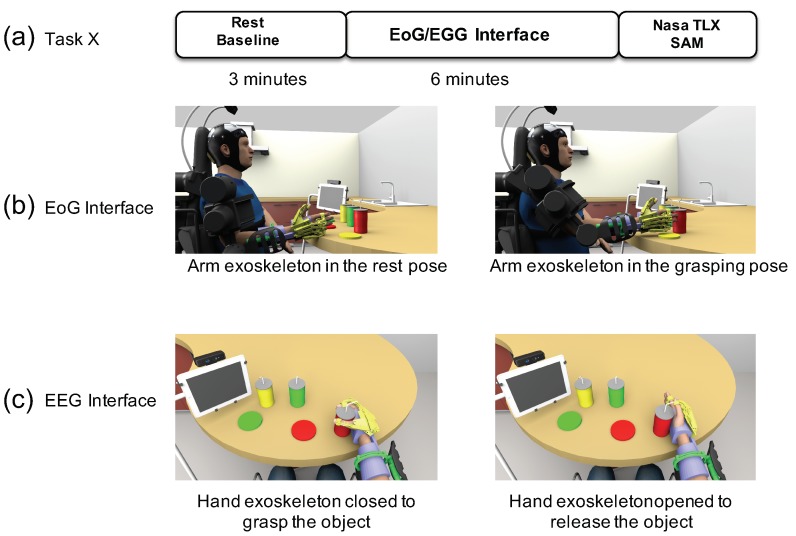
Experimental phase: (**a**) Each subject has to perform two tasks: one of them triggered by EoG interface and the other triggered by EEG interface (each task lasts 6 min). After completing each task, questionnaires were submitted to the user. After completing the questionnaires and before starting with a task, the subjects remain in a relaxed state for 3 min to obtain baseline measurements; (**b**) When the EoG trigger is detected, the exoskeleton moves from rest position to target object position computed by means of the RGB-D camera. Once the object is reached and the EoG trigger is detected, the exoskeleton moves to rest position. (**c**) EEG signal is used to command the closing of the hand exoskeleton. Once the hand is closed, EEG trigger is required to open the hand exoskeleton.

For each participant, both objective and subjective measurements were analysed and quantified. For objective values, some parameters were computed: (i) the performance using each interface; (ii) the activation time computed as the average time to trigger each movement with each interface; and (iii) features extracted from the analysis of the following physicological signals (pulse rate, heart rate variability, respiration rate and galvanic skin response). The subjective measurements were assessed using NASA-TLX and SAM tests after each task as it is shown in Figure 3.

To compare the differences in the EEG and EoG interfaces, different analyses were carried out: correlation analysis to study the evolution of each signal with the time; and a statistical analysis(Mann–Whitney U test) to find differences between both interfaces. All these results are reported in the following subsections.

### 3.1. Interfaces and Performance

As it was explained before, none of the study participants had any prior experience in the use of these kind of interfaces. In the experiment, each participant was asked to perform two different activities using two interfaces: EEG and EoG. Both interfaces were used as a trigger to initiate the movements of the upper arm exoskeleton. Specifically, the activation time was defined as average time to detect a sensorimotor rhythm event-related desynchronization (SMR-ERD) for EEG interface and as average time to detect horizontal eye movements for EoG interface.

In particular, the EoG interface was used to initiate the exoskeleton movement from rest position to target object position computed by means of the RGB-D camera. Once the object is reached and the EoG trigger is detected, the exoskeleton moves to rest position. The mean success rate of EoG interface was 99.85% showing the reliability of the developed biosignal-processing algorithms to detect horizontal eye movements. Additionally, the mean activation time was 1.03 s.

On the other hand, the EEG interface was used to initiate the opening and closing movements of the hand exoskeleton. Once the hand is closed, EEG trigger is required to open the hand exoskeleton. The mean success rate of EEG interface was 63.75%. The mean activation time was 2.2 s.

Finally, the Mann–Whitney U test was used to compare the two interfaces, which showed a very significant difference (p<0.001) for both performance and activation time. In Table 1, the descriptive statistics for both interfaces and results of the Mann–Whitney U test is presented.

### 3.2. Physiological Measurements

Once the physiological signals were acquired, four features were extracted: pulse rate, respiration rate, heart rate variability and skin conductance level. Due to the high intra- and intersubject variability exhibited by psychophysiological responses, all these features were normalized by subtracting the actual value by the baseline value and then dividing the result by the baseline [35].

**Figure 4:**
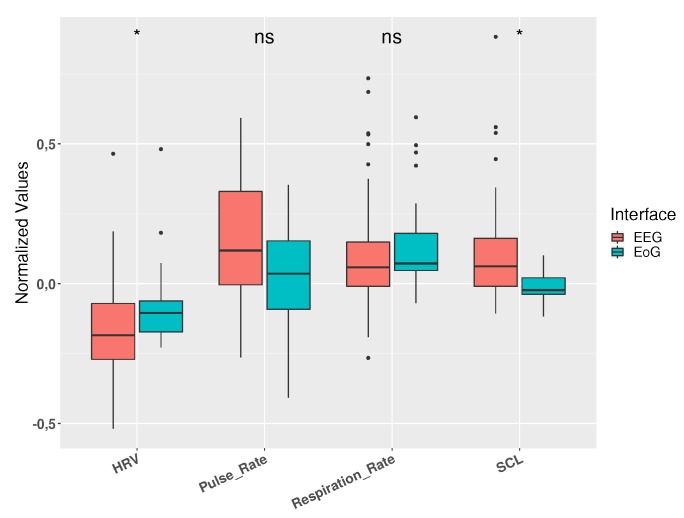
Changes in physiological signals between interfaces. HRV and SCL show a significant difference (* p<0.05) between the two interfaces. Pulse rate shows a trend to be higher in EEG tasks related to EoG tasks.

The Mann–Whitney U test was used to compare the physiological responses between using the two interfaces. In Figure 4, a statistically significant difference (p<0.05) between both interfaces can be shown for both HRV and SCL signals. Pulse rate showed a trend to be higher in EEG tasks related to EoG tasks but the differences were not statistically significant. On the other hand, the respiration rate was very similar from both tasks.

**Figure 5:**
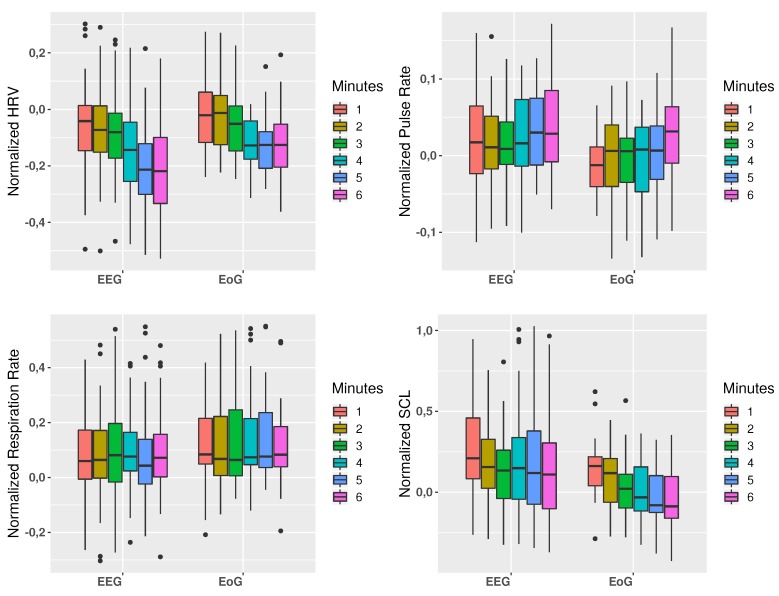
Temporal evolution of each physiological signal (HRV, SCL, respiration and pulse rate). They are shown for each minute (1, 2, 3, 4, 5, and 6 min) and interface (EEG vs. EoG).

In addition, an analysis of the changes over the time for each of the physiological features was performed. For this purpose, both tasks were divided into six periods of one minute in order to evaluate the different signals throughout the complete tasks. In Figure 5, this temporal analysis for both interfaces is presented, observing the mean physiological responses of all subjects during the tasks. Results of this analysis showed a similar behavior in each feature for both interfaces, though the trend in some cases is more pronounced in one of the interfaces. Specifically, these differences are more notable in HRV and SCL signals, where in the first case further decrease is observed for EEG task while in SCL a further decrease is observed for EoG.

Furthermore, a regression and statistical analysis were carried out to study the correlation between the changes in the different features of the physiological signals along the time of the task. In Table 2 results for Pearson correlation coefficient was computed, showing correlations in the decrease of the HRV along the time for both interfaces, as well as a decrease of the SCL for EoG interface. Moreover, the Wilcoxon Signed Rank test was carried out to compare the first and the last minutes for each feature, obtaining statistically significant differences for HRV and SCL for EEG interface and differences for HRV, pulse rate and SCL for EoG interface. These results are shown in the last column of Table 2.

### 3.3. Subjective Ratings

To obtain the subjective perspective of the participants to compare and complete the information obtained with the physiological signals, two different well established questionnaires were completed immediately after each task by each operator: The NASA-TLX [36] and SAM [37] test.

The NASA-TLX is one of the most widely used to assess the subjective workload of the user through the use of six subscales: mental demand, physical demand, own performance, temporal demand, effort, and frustration. First, any possible paired-comparison of the subscales was conducted after each task for each subject. On the other hand, SAM test is an effective measure for self-report emotion recognition. Emotions are rated on a nine-point scale by the two dimensions valence and arousal. Each dimension is represented by nine graphic figures.

**Figure 6:**
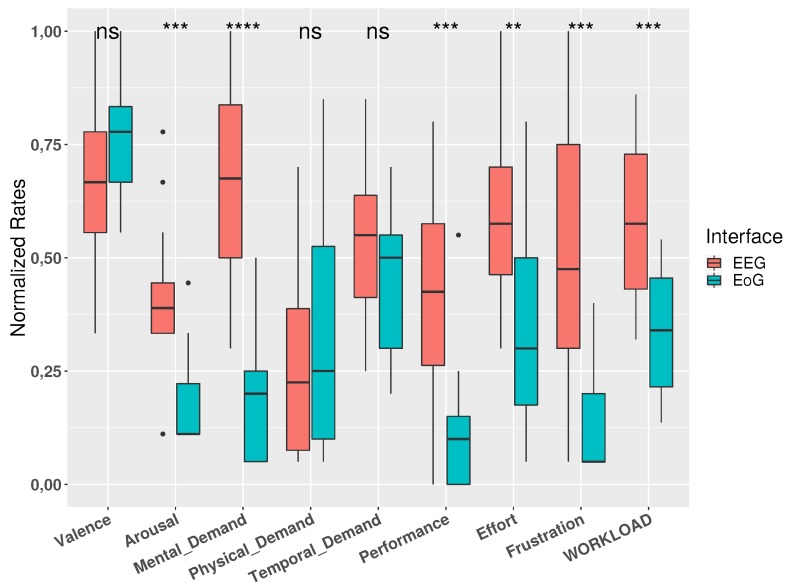
Normalized values of SAM and NASA-TLX tests for both interfaces (** *p* < 0.01; *** *p* < 0.001; **** *p* < 0.0001)

In Table 3 and Figure 6 the data reported by both questionnaires are shown. Additionally, results of a statistical test (Mann-Whitney U test) is also reported in order to compare both interfaces and the subjective perception for the participants. It can be observe that in almost all subjective parameters can be appreciate significant differences between the use of EEG and EoG. Only physical demand and temporal demand from NASA-TLX, and valence from SAM, do not show differences between interfaces; nevertheless, and showing Figure 6, in valence can be observed a slight trend to be higher in the use of EoG. In the same way, temporal demand indicates a slight trend to be higher in the use of EEG. It should be noted that the biggest difference can be observed in the perception of mental demand, much higher in the use of EEG over using EoG

## 4. Discussion

BNCI systems are mainly applied to two scenarios: brain/neural control of assistive devices, such as wearable robots and prostheses, and use of BNCI systems for rehabilitation [24,25]. Lately, the combination of both scenarios, the assistive and rehabilitation ones, was suggested to improve the user acceptability and adoption of BNCI systems [27]. In this context, the optimization of BNCI systems in terms of mental work load and user engagement accessible through the subject’s physiological responses is a subject under investigation in this paper.

Specifically, the aim of our research is to study and analyze the subject’s physiological responses to two different interfaces broadly used in Brain/neural-computer interfaces: EEG and EoG. The analysis of the use of them, and their responses in subject’s physiological state, may lead to understand the best way to use these interfaces during an exhaustive task of hybrid BNCI. Specifically, in this work the subject’s physiological responses are studied and analysed in the case of the use of a hybrid control interface to control an arm/hand exoskeleton for assisting people with disabilities on reaching and grasping objects.

Fatigue is a multidimensional entity, with behavioural, physiological and subjective dimensions, usually related to decreased alertness and increased drowsiness, and is usually accompanied by a reduction in motivation and impairment of task performance. Although mental fatigue is commonly observed following prolonged cognitive activity, its cognitive processes remain largely unknown.

In the literature different signals were demonstrated to be well-established indicators for cognitive workload and/or mental fatigue. Specifically, SCL is a generally well-established indicator of cognitive workload, which increases with mental demand of a task [38]. Furthermore, ECG is usually used as a physiological mark (computing HRV feature) relating its increase with mental fatigue [39,40]. Other studies [41], also suggest the existence of a coherent sequence of changes for EEG, EoG and heart rate variables during the transition from normal drive, high mental workload and eventually mental fatigue and drowsiness.

Our results are in line with these findings, where the temporal analysis shows differences in HRV and SCL from the first minute to the last minute of the tasks. In particular, it can be observe a slight more trend in the decrease of HRV for EoG interface, and a correlation between a decrease of SCL with time only in EoG tasks. Despite motor imagery task should be more intuitive for tasks, such as opening/closing a hand exoskeleton, than EoG, and these results suggest that the higher mental demand due to the motor imagery with respect to the pure physical activity of moving the eyes to one direction cause a higher mental fatigue in the use of EEG interface.

In addition, our results showed changes in HRV and SCL from the first to the last minutes of the task for each interface. There are statistically significant differences for HRV and SCL for EEG interface and differences for HRV, pulse rate and SCL for EoG interface. These findings could be the first step to adapt BNCI systems using changes in physiological reactions(e.g to dynamically adapt the bio-signal detection thresholds, etc.).

Regarding subjective rates, the differences in physiological signals between the use of both interfaces are consistent with the perceived workload resulting from NASA-TLX test.

These results suggest possible frustration and stress due to a long-time use of Hybrid BNCI. Since few studies [19,20,21] associate the user’s psychological state with a performance decrease in brain-computer interface, understanding these physiological responses may lead us to develop adaptive techniques to detect and minimize this stress induced to the user by, for instance, changing between interfaces and using the most intuitive or the lesser mental demand one.

Furthermore, regression and statistical analyses were carried out to study the correlation between the changes in the different features of the physiological signals along the time of the task. In Table 2 results for Pearson correlation coefficient was computed, showing correlations in the decrease of the HRV along the time for both interfaces, as well as a decrease of the SCL for EoG interface. Moreover, Wilcoxon Signed Rank test was carried out to compare the first and the last minutes for each feature, obtaining statistically significant differences for HRV and SCL for EEG interface and differences for HRV, pulse rate and SCL for EoG interface. These results can be shown in the last column of Table 2.

## 5. Conclusions

Our main finding in this work is that the exhaustive use of BNCI interfaces produce subjects’ quantitative changes in the physiological reactions in that subjects, and these changes are different for different interfaces: EEG and EoG. Specifically, our results suggest that HRV and SCL show significant differences between good and poor performance using both interfaces. Furthermore, the temporal analysis performed to the four physiological features processed, states a relationship between the amount of time exhaustively using the interfaces with the changes in the physiological response of the subjects. These analyses show a correlation between time and a decrease in HRV for both interfaces, which can indicate an increase in the level of stress of the subject’s psychological state due to the exhaustiveness of the tasks. In addition, a correlation between time and a decrease in SCL is appreciated in the use of EoG, which can be explained with the lack of mental demand in the used of this interface.

In addition, our measured results are in accordance with the results of subject’s workload perception and emotional responses assessed through NASA-TLX questionnaires and Self-Assessment Manikin(SAM) respectively.

This is the first step to adapt our Hybrid BNCI using changes in physiological reactions to customise the use of different interfaces during a long-time use of Hybrid BNCI, for instance changing between interfaces, with the aim of avoiding possible frustration and stress due to the high mental demand in EEG interfaces, which may lead to a decrease in the subject’s performance.

## Figures and Tables

**Table 1:** Descriptive statistics and results of Mann-Whitney U test between interfaces.

	Performance		Activation Time	
	**EEG**	**EoG**	**EEG**	**EoG**
**Min.**	21.43	96.77	1.55	0.85
**1st Qu.**	51.01	100.00	1.92	0.94
**Median**	65.71	100.00	2.10	1.02
**Mean**	63.75	99.85	2.20	1.03
**3rd Qu.**	79.09	100.00	2.46	1.11
**Max.**	93.75	100.00	3.20	1.25
**Skewness**	−0.58	−4.07	0.61	0.32
**Kurtosis**	−0.74	15.27	−0.44	−0.71
**Mann-Whitney test**		
***p*** **-value**	<0.001		<0.001	

**Table 2:** Temporal statistical results of the physiological signals: Pearson correlation between each signal and time is shown; as well as Wilcoxon Signed Rank test comparing the first and the last minute of each task.

		Pearson Correlation			Wilcoxon Signed Rank Test
		(Signal vs. Time)			(Minute 1 vs. Minute 6)
		**rho**	$R^{2}$	***p*** **-Value**	***p*** **-Value**
	HRV	−0.344	0.118	<0.001	<0.001
**EEG**	Pulse Rate	0.134	0.018	0.030	0.064
	Resp. Rate	0.011	0.0001	0.865	0.544
	SCL	−0.086	0.007	0.234	<0.001
	HRV	−0.339	0.115	0.001	0.011
**EoG**	Pulse Rate	0.156	0.024	0.087	0.007
	Resp. Rate	0.016	0.0001	0.868	0.920
	SCL	−0.306	0.094	0.001	0.001

**Table 3:** Report of questionnaires (mean ± STD) and statistical results of Mann-Whitney U test: all but three parameters (physical demand, temporal demand and valence) show significant differences between both interfaces.

Parameter	EEG		EoG		Mann-Whitney U Test
	Ratings	Scales	Ratings	Scales	*p*-Value
Mental Demand	13.23 ± 4.06	3.82 ± 2.86	4.05 ± 1.03	3.00 ± 1.15	<0.0001
Physical Demand	4.95 ± 3.56	6.55 ± 5.32	0.64 ± 0.89	3.36 ± 1.26	0.54
Temporal Demand	10.68 ± 2.99	8.82 ± 3.40	2.55 ± 1.35	3.10 ± 1.34	0.19
Performance	8.68 ± 4.10	2.36 ± 3.24	2.10 ± 0.86	0.45 ± 0.80	<0.001
Effort	12.00 ± 3.54	7.18 ± 4.55	2.91 ± 1.05	3.73 ± 1.08	0.007
Frustration	10.45 ± 5.34	2.73 ± 2.27	2.77 ± 1.70	1.36 ± 0.79	<0.001
Workload	58.38 ± 16.52		33.82 ± 14.78		<0.001
Valence	5.95 ± 1.56		6.82 ± 1.40		0.14
Arousal	3.95 ± 1.56		1.73 ± 1.01		<0.001

## References

[1] European Commision (2010). People with disabilities have equal rights. The European Disability Strategy 2010–2020.

[2] Müller-Putz G.R., Breitwieser C., Cincotti F., Leeb R., Schreuder M., Leotta F., Tavella M., Bianchi L., Kreilinger A., Ramsay A. (2011). Tools for brain–computer interaction: A general concept for a hybrid BCI. Front. Neuroinform..

[3] Choi I., Rhiu I., Lee Y.S., Yun M.W., Nam C.S. (2017). A Systematic Review of Hybrid Brain-Computer Interfaces: Taxonomy and Usability Perspectives. PLoS ONE.

[4] Su Y., Qi Y., Luo J.X., Wu B., Yang F., Li Y., Zhuang Y.T., Zheng X.X., Chen W.D. (2011). A hybrid brain-computer interface control strategy in a virtual environment. J. Zhejiang Univ. Sci. C.

[5] Pan J., Xie Q., He Y., Wang F., Di H., Laureys S., Yu R., Li Y. (2014). Detecting awareness in patients with disorders of consciousness using a hybrid brain-computer interface. J. Neural Eng..

[6] Allison B.Z., Brunner C., Kaiser V., Muller-Putz G.R., Neuper C., Pfurtscheller G. (2010). Toward a hybrid brain computer interface based on imagined movement and visual attention. J. Neural Eng..

[7] Úbeda A., Iáñez E., Badesa J., Morales R., Azorín J.M., García N. Control strategies of an assistive robot using a Brain-Machine Interface. Proceedings of the 2012 IEEE/RSJ International Conference on Intelligent Robots and Systems.

[8] Yu T., Xiao J., Wang F., Zhang R., Gu Z., Cichocki A., Li Y. (2015). Enhanced motor imagery training using a hybrid BCI with feedback. IEEE Trans. Biomed. Eng..

[9] Brunner C., Allison B.Z., Krusienski D.J., Mullerputz G.R., Pfurtscheller G., Neuper C. (2010). Improved signal processing approaches in an offline simulation of a hybrid brain-computer interface. J. Neurosci. Methods.

[10] Li X., Samuel O.W., Zhang X., Wang H., Fang P., Li G. (2017). A motion-classification strategy based on sEMG-EEG signal combination for upper-limb amputees. J. Neuroeng. Rehabil..

[11] Kawase T., Sakurada T., Koike Y., Kansaku K. (2017). A hybrid BMI- based exoskeleton for paresis: EMG control for assisting arm movements. J. Neural Eng..

[12] Scherer R., Mller-Putz G.R., Pfurtscheller G. (2007). Self-initiation of eeg-based brain-computer communication using the heart rate response. J. Neural Eng..

[13] Pfurtscheller G., Allison B.Z., Brunner C., Bauernfeind G., Escalante T.S., Scherer R., Zander T.O., Mueller-Putz G., Neuper C., Birbaumer N. (2010). The hybrid bci. Front. Neurosci..

[14] Witkowski M., Cortese M., Cempini M., Mellinger J., Vitiello N., Soekadar S.R. (2014). Enhancing brain–machine interface (BMI) control of a hand exoskeleton using electrooculography (EoG). J. Neuroeng. Rehabil..

[15] Surjo R. (2015). Soekadar, Matthias Witkowski, Nicola Vitiello, Niels Birbaumer An EEG/EoG-based hybrid brain-neural computer interaction (BNCI) system to control an exoskeleton for the paralyzed hand. Biomed. Tech..

[16] Frisoli A., Procopio C., Chisari C., Creatini I., Bonfiglio L., Bergamasco M., Rossi B., Carboncini M. (2012). Positive effects of robotic exoskeleton training of upper limb reaching movements after stroke. J. NeuroEng. Rehabil..

[17] Barsotti M., Leonardis D., Loconsole C., Solazzi M., Sotgiu E., Procopio C., Chisari C., Bergamasco M., Frisoli A. A full upper limb robotic exoskeleton for reaching and grasping rehabilitation triggered by MI-BCI. Proceedings of the 2015 IEEE International Conference on Rehabilitation Robotics (ICORR).

[18] Pedrocchi A., Ferrante S., Ambrosini E.G., Olla M., Casellato C., Schauer T., Klauer C., Pascual J., Vidaurre C., Gfohler M. (2013). Mundus project: MUltimodal neuroprosthesis for daily upper limb support. J. Neuroeng. Rehabil..

[19] Kaufmann T., Vögele C., Sütterlin S., Lukito S., Kübler A. (2012). Effects of resting heart rate variability on performance in the P300 brain-computer interface. Int. J. Psychophysiol..

[20] Myrden A., Chau T. (2015). Effects of user mental state on EEG-BCI performance. Front. Hum. Neurosci..

[21] Myrden A., Chau T. (2017). A Passive EEG-BCI for Single-Trial Detection of Changes in Mental State. IEEE Trans. Neural Syst. Rehabil. Eng..

[22] Mohammadpour M., Mozaffari S. Classification of EEG-based attention for brain computer interface. Proceedings of the 2017 3rd Iranian Conference on Intelligent Systems and Signal Processing (ICSPIS).

[23] Li Y., Li X., Ratcliffe M., Liu L., Qi Y., Liu Q. A real-time EEG-based BCI system for attention recognition in ubiquitous environment. Proceedings of the 2011 International Workshop on Ubiquitous Affective Awareness and Intelligent Interaction (UAAII ’11).

[24] Ushiba J., Soekadar S.R. (2016). Brain-machine interfaces for rehabilitation of poststroke hemiplegia. Prog. Brain Res..

[25] Soekadar S.R., Birbaumer N., Slutzky M.W., Cohen L.G. (2015). Brain-Machine Interfaces in Neurorehabilitation of Stroke. Neurobiol. Dis..

[26] Soekadar S.R., Witkowski M., Gómez C., Opisso E., Medina J., Cortese M., Cempini M., Carrozza M.C., Cohen L.G., Birbaumer N. (2016). Hybrid EEG/EoG-based brain/neural hand exoskeleton restores fully independent daily living activities after quadriplegia. Sci. Robot..

[27] Soekadar S.R., Nann M., Crea S., Trigili E., Gómez C., Opisso E., Cohen L.G., Birbaumer N., Vitiello N., Guger C., Mrachacz-Kersting N., Allison B. (2019). Restoration of Finger and Arm Movements Using Hybrid Brain/Neural Assistive Technology in Everyday Life Environments. Brain-Computer Interface Research.

[28] Crea S., Nann M., Trigili E., Cordella F., Baldoni A., Badesa F.J., Catalán J.M., Zollo L., Vitiello N., Aracil N.G. (2018). Feasibility and safety of shared EEG/EOG and vision-guided autonomous whole-arm exoskeleton control to perform activities of daily living. Scienfic Rep..

[29] Pfurtscheller G., da Silva L.F.H. (1999). Event-related EEG/MEG synchronization and desynchronization: Basic principles. Clin. Neurophysiol..

[30] Toyama S., Takano K., Kansaku K. (2012). A non-adhesive solid-gel electrode for a non-invasive brain-machine interface. Front. Neurol..

[31] McFarl D.J. (2015). The advantages of the surface Laplacian in brain-computer interface research. Int. J. Psychophysiol..

[32] Crea S., Cempini M., Moisè M., Baldoni A., Trigili E., Marconi D., Cortese M., Giovacchini F., Posteraro F., Vitiello N. A novel shoulder-elbow exoskeleton with series elastic actuators. Proceedings of the 6th IEEE International Conference on Biomedical Robotics and Biomechatronics (BioRob).

[33] Trigili E., Crea S., Moisè M., Baldoni A., Cempini M., Ercolini G., Marconi D., Posteraro F., Carrozza M.C., Vitiello N. (2019). Design and Experimental Characterization of a Shoulder-Elbow Exoskeleton with Compliant Joints for Post-Stroke Rehabilitation. IEEE/ASME Trans. Mechatron..

[34] Díez J.A., Blanco A., Catalán J.M., Badesa F.J., Lledó L.D., García-Aracil N. (2018). Hand exoskeleton for rehabilitation therapies with integrated optical force sensor. Adv. Mech. Eng..

[35] Novak D., Mihelj M., Munih M. (2012). A survey of methods for data fusion and system adaptation using autonomic nervous system responses in physiological computing. Interact. Comput..

[36] NASA (1986). Nasa Task Load Index (TLX) v. 1.0 Manual.

[37] Bradley M.M., Lang P.J. (1994). Measuring emotion: The self-assessment manikin and the semantic differential. J. Behav. Ther. Exp Psychiatry.

[38] Collet C., Averty P., Dittmar A. (2009). Autonomic nervous system and subjective ratings of strain in air-traffic control. Appl. Ergon..

[39] Egelund N. (1982). Spectral analysis of heart rate variability as an indicator of driver fatigue. Ergonomics.

[40] Mascord D.J., Heath R.A. (1992). Behavioral and physiological indices of fatigue in a visual tracking task. J. Saf. Res..

[41] Borghini G., Astolfi L., Vecchiato G., Mattia D., Babiloni F. (2014). Measuring neurophysiological signals in aircraft pilots and car drivers for the assessment of mental workload, fatigue and drowsiness. Neurosci. Biobehav. Rev..

